# Detecting position dependent tremor with the Empirical mode decomposition

**DOI:** 10.1186/s40734-014-0014-z

**Published:** 2015-02-16

**Authors:** André Lee, Eckart Altenmüller

**Affiliations:** Institute of Music Physiology and Musicians’ Medicine, Hannover University of Music, Drama and Media, Emmichplatz 1, 30175 Hannover, Germany

**Keywords:** Essential tremor, Dystonia, Dystonic tremor, EMD, Hilbert spectrum, Musician

## Abstract

**Background:**

Primary bowing tremor (PBT) occurs in violinists in the right bowing-arm and is a highly nonlinear and non-stationary signal. However, Fourier-transform based methods (FFT) make the a priori assumption of linearity and stationarity. We present an interesting case of a violinist with PBT and apply a novel method for nonlinear and non-stationary signals for tremor analysis: the empirical mode decomposition (EMD). We compare the results of FFT and EMD analyses.

**Methods:**

Tremor was measured and quantified in a 50-year-old professional violinist with an accelerometer. Data were analyzed using the EMD, the Hilbert transform, the Hilbert spectrum and the marginal Hilbert spectrum. Findings are compared to the FFT-spectrum and FFT-spectrogram.

**Results:**

We could show that the EMD yields intrinsic mode functions, which represent the tremor and IMFs, which are associated with voluntary movement. The instantaneous frequency and amplitude are obtained. In contrast the low time frequency resolution and the artifacts of voluntary movements are seen in the FFT results.

**Conclusions:**

PBT may present itself as a highly non-stationary and nonlinear phenomenon, which can be accurately analyzed with the EMD, since it gives the instantaneous amplitude and frequency and can identify voluntary from involuntary (tremor) movement.

**Electronic supplementary material:**

The online version of this article (doi:10.1186/s40734-014-0014-z) contains supplementary material, which is available to authorized users.

## Background

Tremor is defined as an involuntary rhythmical oscillation of a body part [1]. Particularly pathological tremors are time-varying [2] and highly nonlinear and non-stationary in nature [1,3,4]. Task-specific tremors (TST) are pathological tremors that occur predominantly during certain tasks [1]. Primary bowing tremor (PBT) [5] occurs unilaterally in the right arm of bowed string-instrument players while playing the instrument. This is a highly disabling condition and may threaten the musician’s professional career. We describe a violinist in whom PBT occurred when he played a fast movement from the tip of the bow to the frog (the part of the bow held by the violinist), brought the movement to a sudden stop and tried to maintain the hand in a stable position (Additional file 1: Video). In that position tremor appeared and decreased in amplitude over the next 10–20 seconds, giving a highly non-stationary and non-linear signal.

The disadvantage of applying the Fourier transform (FFT) to these kinds of signals is the *a-priori* assumption of a linear and periodic or stationary signal i.e. a sine or cosine of constant amplitude and frequency spanning the whole signal. The FFT gives reliable results therefore only in case of linear and stationary signals [6]. However, periodicity cannot be assumed for tremors, since frequency not only changes with the waves in a dispersive system (interwave modulation) but likewise within one oscillation cycle or wave (intrawave modulation) [7]. Therefore the wave-profile cannot be considered a sine or cosine function. Furthermore FFT has a limited time-frequency resolution. Thus potentially meaningful local (in a temporal sense) oscillations may not be detected. Finally the FFT does not distinguish between noise (e.g. voluntary movement in this study) and the actual signal (e.g. tremor, as in this study), making the result less reliable. In recent studies a new method that takes into account the nonlinearity and non-stationarity of signals has been introduced [8] and has been applied in tremor research [2,9,10]. This approach combines two tools: Empirical mode decomposition (EMD) and the Hilbert transform.

**Figure 1:**
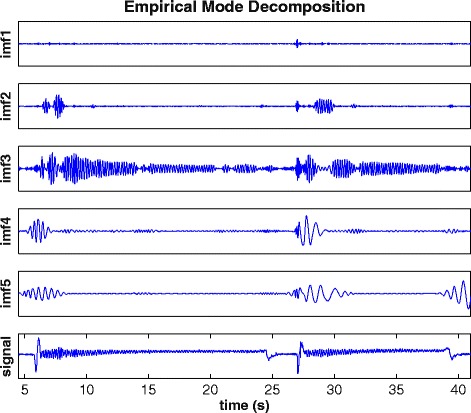
**Empirical mode decomposition of the original signal with the intrinsic mode functions (imf) 1–5 and the original signal at the bottom.** The x-axis displays the time in seconds. Details are described in the text.

EMD decomposes signals into basic components, called intrinsic mode functions (IMFs, Figure 1). In contrast to the FFT, the EMD is an adaptive, data-driven, *a-posteriori* approach, which does not need a-priori assumptions with regard to the signal [7,8]. The IMF can be regarded as a more general counterpart of the simple harmonic functions obtained by the FFT that may have a variable amplitude and frequency [7]. It has been shown that IMFs can be used to identify distinct frequency bands associated with physical or physiological phenomena, for example particular types of tremor, [9,11,12] however it has been noted that mainly tremor data from gyroscopes was analyzed [9,11,12], but not from accelerometers. Furthermore, the EMD may separate noise from the actual data [8].

The Hilbert transform yields the Hilbert spectrum, i.e. the instantaneous amplitude and frequency. It is thus a measure of the contribution of each frequency over time, from which the marginal Hilbert spectrum (MHS), a measure of the total amplitude contribution for each frequency value [7,8], can be derived. The advantage of this method over the FFT is that it is a windowing independent time-frequency representation with a high time-frequency resolution.

The aim of this paper was thus threefold: 1. to present an interesting case of a task-specific tremor in a violinist; 2. to investigate, whether the EMD and Hilbert transform can identify the tremor signal from the highly non-stationary and nonlinear signal obtained from the accelerometer and to separate artifacts from voluntary movements inherent to the task from the involuntary tremor (see Methods) 3. to demonstrate the advantages of the EMD over the FFT.

## Methods

The study was approved by the ethics committee of the Hanover medical school and written informed consent was obtained by the participant. Tremor was measured in a 50-year-old professional violinist who had played in a prestigious orchestra for more than 20 year. He was asked to play a fast up-bow-movement, which triggered tremor at the end of that movement when trying to hold the hand in a stable position. He then took back the bow from the frog of the bow to the tip to prepare another fast up-bow movement. The fast up-bow movement and retaking the bow are referred to as voluntary movement. This was repeated for five times. Measurement occurred with a 3D accelerometer (biovision, Wehrheim, Germany, 8×8×11 mm; 4 gram; DC–500 Hz; max 50 g), which was attached to the metacarpo-phalangeal (MCP) joint of the index finger of the right hand. Data were bandpass filtered using a 4th degree butterworth-filter (cutoff 1–50 Hz), applied back and forth to compensate for phase shift. With the accelerometer signal onset and end of the fast up-bow movement as well as retaking the bow could be identified.

### Empirical mode decomposition

The EMD consists of a sifting process of the original signal [X(t)], in which the intrinsic mode functions (IMF) are obtained. The EMD algorithm as described by Huang et al. is as follows [7,8].

An envelope is created by connecting the local maxima and minima of X(t) with a cubic spine interpolation. The mean value is calculated by taking the average of the upper and lower limit of the envelope (m_1_) and subtract it from X(t):

1 $$ X(t)-{m}_1(t)={h}_1(t) $$

In order to be considered an IMF two conditions must be fulfilled: 1) the number of extrema and the number of zero crossings must be either equal or differ at most by one, and 2) at any time the mean value of the envelope defined by the local maxima and the envelope defined by the local minima is zero [7,8]. If h_1_(t) does not fulfill the criteria for an IMF, this procedure is repeated n-times until h_(1n)_(t) fulfills the criteria and is thus defined as the first IMF [c_1_(t)]:

2 $$ {c}_1(t)={h}_{1n}(t) $$

Next, c_1_(t) is subtracted from X(t):

3 $$ X(t)-{c}_1(t)={r}_1(t) $$

r_1_(t) is called the residue which is substituted for X(t) in formula (1) and the first steps are repeated m-times until a residual r_m_ is reached that is a monotonic function of which no more IMFs can be extracted [7,8]. Thus, the original signal can be obtained by

4 $$ X(t)={\displaystyle \sum_{i=1}^m}{c}_i(t)+{r}_m $$

The frequency ranges of the IMF are ordered in such a way that IMF1 contains the highest and IMF_m_ the lowest frequencies. Being a data-driven approach, the frequency ranges depend on the original signal.

### Hilbert transform, Hilbert spectrum and marginal Hilbert spectrum

The Hilbert transform is applied to the IMFs to obtain the instantaneous frequency and amplitude (Hilbert-spectrum) i.e. the amplitude and frequency at each moment in the movement. The MHS h(ω) is obtained by

5 $$ h\left(\omega \right)={\displaystyle \underset{0}{\overset{T}{\int }}}H\left(\omega, t\right)dt $$

where H(ω,t) is the Hilbert spectrum derived by

6 $$ H\left(\omega, t\right)={\displaystyle \sum_{j=1}^n}{a}_j(t)\  \exp \left[i{\displaystyle \int }{\omega}_j(t)dt\right] $$

a_j_(t) is the instantaneous amplitude, ω_j_(t) the instantaneous frequency.

A detailed description can be found in Huang et al. [8] and Huang [7].

We expected to find an IMF, that would display the onset of tremor after stopping the up-bow movement with a decreasing amplitude without the artifact of the fast up-bow movement itself.

EMD was performed in Matlab using the EMD package by Rilling et al. [http://perso.ens-lyon.fr/patrick.flandrin/emd.html], applying the default stopping criterion [13].

### FFT for comparison

To compare the results of the EMD and Hilbert transform with the FFT we performed an FFT with a window of 5096 data points and an overlap of 1024 data points.

## Results

### Tremor detection

#### Empirical mode decomposition

Figure 1 displays the EMD with 5 IMFs and the original signal for the first two fast up-bow movements. In the original signal the artifact voluntary movements (i.e. at the onset of the fast up-bow movement and when taking back the bow) are visible, showing the non-stationarity of the signal. IMF3 best represents the tremor signal, however, part of signal becomes apparent in IMF2, known as mode-mixing. No tremor is detected in IMF1. Low-frequency tremor can be seen in IMFs 4 and 5. To investigate the mode mixing we chose the combination of IMF2 and IMF3 (IMF2 + 3) for further evaluation (see below).

**Figure 2:**
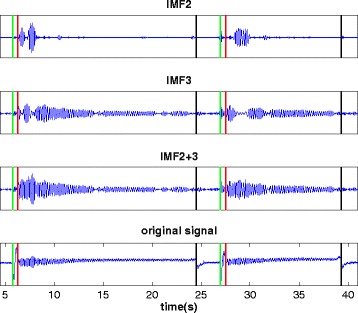
**Depicted are IMF2, IMF3, the combination of IMF2 plus IMF3 = IMF2 + 3 and the original signal.** The x-axis shows the time in seconds. The vertical green line shows the onset and the vertical red line the end of the fast up-bow movement as identified from the acceleration signal in the original signal. The vertical black line shows the moment, when the bow is retaken from the frog of the bow to the tip for the second fast up-bow movement. One fast up-bow movement took about 0.5 seconds, whereas retaking the bow took about 2 s.

Figure 2 displays IMF2 and IMF3, the combination of both, IMF2 + 3, as well as the original signal. IMF2 + 3 give a more accurate representation of the tremor signal. In the IMFs and the original signal the moments where voluntary movements occur are indicated by vertical lines (see legend).

#### Hilbert spectrum and marginal Hilbert spectrum

**Figure 3:**
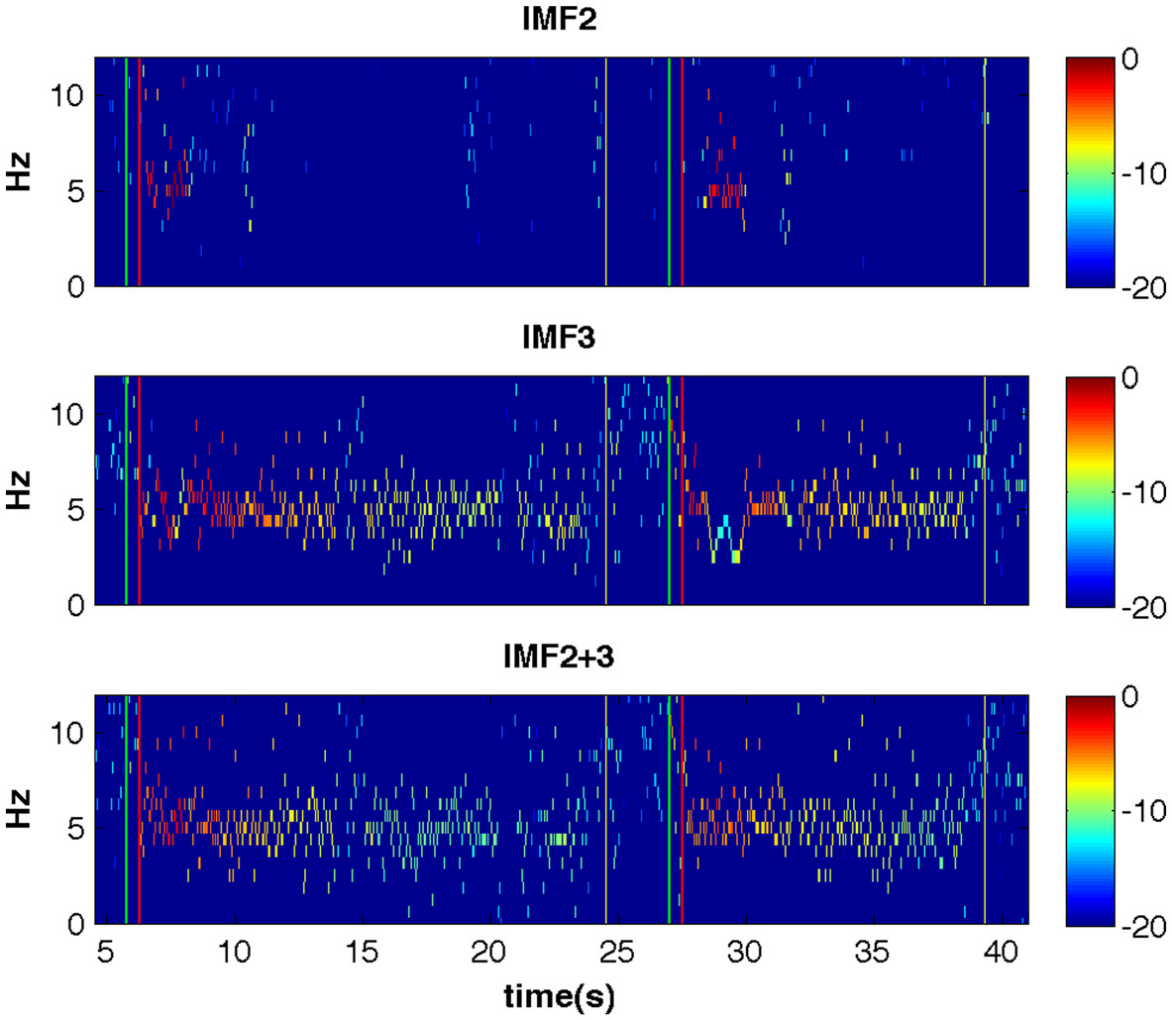
**Hilbert spectrum of IMF2, IMF3 and IMF2 + 3.** The x-axis shows the time in seconds. The vertical green and red line are as in Figure 2, for visibility reasons the vertical black line of Figure 2 is shown in yellow here. The instantaneous frequency can be seen around 5 Hz. The amplitude of the tremor is color-coded; the colorbar units are dB. The onset of tremor after stopping the bow (vertical red line) and the decrease of amplitude until the bow is taken back to the tip (vertical yellow line) can be clearly seen and is best represented in IMF2 + 3. Higher frequency of low amplitude can be seen when the bow is taken back (between the vertical yellow and green line).

**Figure 4:**
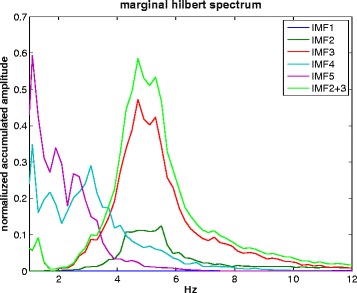
**Marginal Hilbert spectrum of IMF1 – IMF5 and IMF2 + 3.** IMF1 is not visible due to the large scale. The main contribution of IMF3 to tremor-detection becomes visible and the mode mixing of IMF2 + 3 does not alter the peak frequency.

Figure 3 shows the Hilbert spectrum i.e. the time-frequency representation of IMF2, IMF3 and IMF2 + 3. The instantaneous frequency and amplitude are shown, as described in the methods part. Figure 4 depicts the MHS of IMFs 1 to 5 as well as IMF2 + 3. The peak amplitude of both, IMF3 and IMF2 + 3 is 4.7 Hz. Lower frequencies are detected in IMF4 and IMF5.

### Comparison with FFT

**Figure 5:**
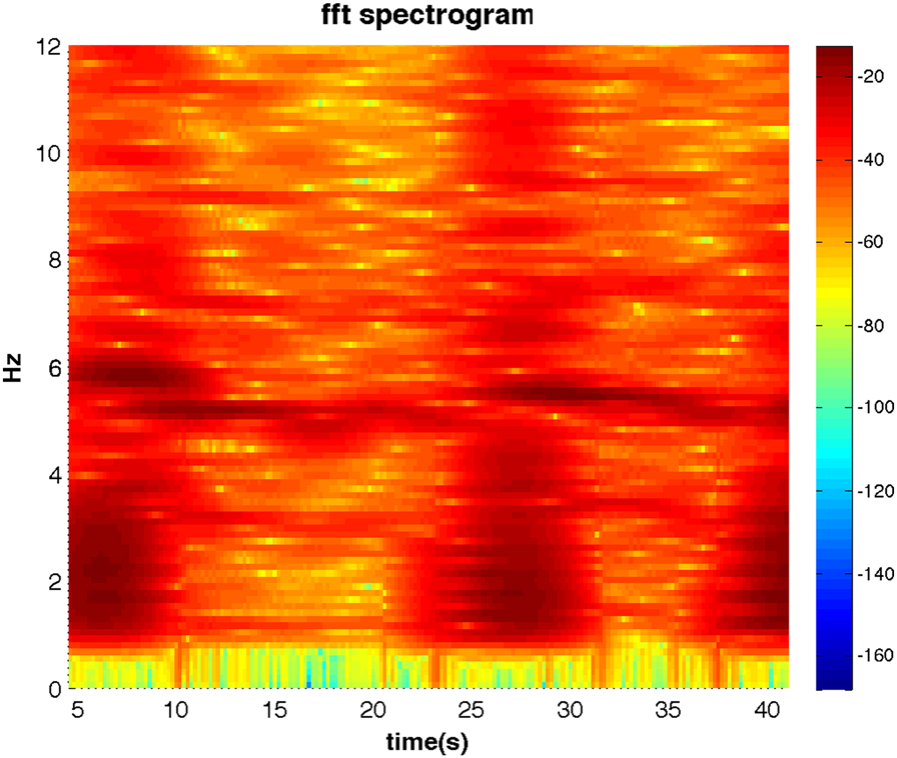
**The spectrogram of the FFT.**

**Figure 6:**
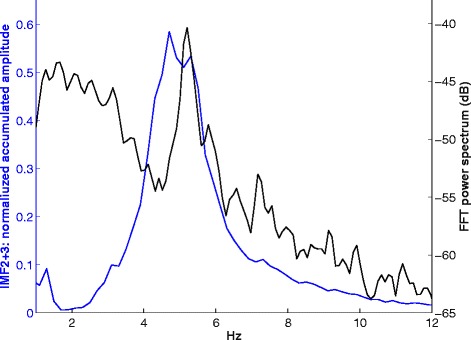
**The FFT power spectrum of the original signal (black), with the legend on the right y-axis in dB.** The MHS of IMF2 + 3 (blue) with the legend on the left y-axis in normalized accumulated amplitude. The x-axis shows the frequency in Hz. for both graphs.

Figure 5 depicts the FFT-spectrogram of the original signal. The low time-frequency resolution becomes apparent. Figure 6 depicts the FFT power spectrum of the original signal with a peak amplitude at 5.2 Hz and the MHS of IMF2 + 3 for comparison.

## Discussion

We describe a patient with an unusual form of task specific bowing tremor that appeared only when a sudden up-bow movement was brought to a sudden stop at the frog of the bow and held there as steadily as possible. The peak frequency was at 4.7 Hz (Figure 4) and thus was in the range of PBT described before [14,15]. Since we had a highly non-stationary signal with artifacts from voluntary movements (i.e. the fast up-bow movement as well as the movement for returning the bow to the tip) as seen in Figures 1 and 2, we applied a novel method for analyzing the signal, the EMD and Hilbert transform that do not require stationarity and linearity as a prerequisite. We could show that the EMD and the Hilbert transform are able to correctly identify the tremor signal. As expected the tremor signal was mainly contained in one IMF, namely IMF3. One interesting aspect of our analysis was the finding of a mode mixing, which has been described by Huang [7]. It describes the finding of part of the tremor signal being distributed between two (or more) IMFs, in our case between IMF2 and IMF3. According to formula (4), the original signal can be obtained by adding all IMF and the final residual r_m_, which implies the possibility of adding two (or more) IMF. We therefore added IMF2 + IMF3. The Hilbert spectrum of this combined IMF2 + 3 (Figure 3, bottom) corroborates this finding, since the tremor amplitude of very precisely represents the course of the tremor observed during clinical examination at the instrument (Additional file 1: Video): It is highest after the up-bow movement and diminishes over time. Virtually no tremor is present when retaking the bow.

Importantly, our expectation that the EMD can reduce artifacts, e.g. from voluntary movement as has been shown before [9], was confirmed. As shown in Figure 2, the low frequency artifacts of the up-bow movement and of retaking the bow are removed in the IMFs. The up-bow movement lasted for 0.5 s, giving a low frequency artifact of 2 Hz. Even lower frequencies were to be expected from retaking the bow. Peaks at around 2 Hz and below are visible in IMF4 and IMF5. We therefore interpret the low frequency signal of these IMFs (Figure 1) to be caused by the voluntary movements. This is important, because it suggests that discontinuities in tremor signals and voluntary movements have a small effect on the precision of the method.

Given the algorithm of the EMD, fast voluntary movements in music making with a high frequency (e.g. tremolo) would have been represented in IMF 1. Our paradigm did not include those kinds of movements and thus, as expected, almost no signal was detected here.

The comparison with results of an FFT analysis revealed similar peak frequencies for both methods. However, the limited time-frequency resolution of the FFT becomes apparent when comparing Figures 3 and 5. The instantaneous frequency and amplitude allows a more precise course of the tremor, whereas for FFT-based methods, a compromise between time and frequency resolution has to be made. In the FFT spectrogram (Figure 6) the low-frequency artifact of the voluntary movement is overestimated and taken into account in the FFT power spectrum (Figure 5). In the IMFs, however, the signal is separated into frequency ranges that cover both, tremor (IMF2 + 3) and lower frequency voluntary movement (IMF 4, IMF5, see Figure 4). We therefore conclude that IMF2 + 3 represents the frequency range and thus the intrawave frequency modulation of task-specific tremor than the frequency range covered by the FFT power spectrum (Figure 5). This is important, because it has been shown that the wave-profile deformation seen in the FFT-power spectrum that are usually interpreted as harmonic distortions, are more likely due to the intrawave frequency modulation [8].

Since the EMD has been applied so far only for tremor data derived from a gyroscope [2,9,10] we finally conclude that it is applicable for accelerometer data, as well.

## Conclusion

We present an interesting and unusual case of a patient with a highly non-stationary and nonlinear PBT. We could show that the EMD can accurately detect and analyze the tremor, identify voluntary movement and is applicable for data obtained with an accelerometer. In comparison to the FFT, the EMD does not make a priori assumptions on the data, yields the instantaneous frequency and amplitude and is thus a more precise tool for analyzing tremor data.

## Additional files

Additional file 1: Video. The bowing arm of the patient. The tremor appears after bringing the fast upward movement of the bow to a stop and trying to hold the arm in a steady position. The amplitude decreases with time. Please note that at second 14 the video is zoomed in and at second 18 zoomed out again so that the patient cannot be identified. The sound however is continuous.

## References

[1] Deuschl G, Bain P, Brin M (1998). Consensus statement of the Movement Disorder Society on Tremor. Ad Hoc Scientific Committee. Mov Disord.

[2] De Lima ER, Andrade AO, Pons JL, Kyberd P, Nasuto SJ (2006). Empirical mode decomposition: a novel technique for the study of tremor time series. Med Biol Eng Comput.

[3] Gantert C, Honerkamp J, Timmer J (1992). Analyzing the dynamics of hand tremor time series. Biol Cybern.

[4] Timmer J, Haussler S, Lauk M, Lucking C-H (2000). Pathological tremors: deterministic chaos or nonlinear stochastic oscillators?. Chaos.

[5] Lee A, Altenmüller E (2012). Primary task-specific tremor: an entity of its Own?. Med Probl Perform Art.

[6] Oppenheim AV, Schafer RW (1989). Discrete-Time Signal Processing.

[7] Huang NE (2005). Introduction to the Hilbert–Huang Transform and Its Related Mathematical Problems. Hilbert-Huang Transform and its Applications.

[8] Huang NE, Shen Z, Long SR, Wu MC, Shih HH, Zheng Q, Yen N-C, Tung CC, Liu HH (1998). The empirical mode decomposition and the Hilbert spectrum for nonlinear and non-stationary time series analysis. Proc R Soc A: Math Phys Eng Sci.

[9] Rocon E, Pons JL, Andrade AO, Nasuto SJ. Application of EMD as a novel technique for the study of tremor time series. Conf Proc IEEE Eng Med Biol Soc. 2006, Suppl:6533–6536. 10.1109/IEMBS.2006.260871 17959445

[10] Gallego JA, Rocon E, Koutsou AD, Pons JL. Analysis of kinematic data in pathological tremor with the Hilbert-Huang transform. IEEE. 2011:80–83

[11] Silchenko AN, Adamchic I, Pawelczyk N, Hauptmann C, Maarouf M, Sturm V, Tass PA (2010). Data-driven approach to the estimation of connectivity and time delays in the coupling of interacting neuronal subsystems. J Neurosci Methods.

[12] Li K, Hogrel J-Y, Duchêne J, Hewson DJ (2011). Analysis of fatigue and tremor during sustained maximal grip contractions using Hilbert-Huang Transformation. Med Eng Phys.

[13] Hogan N (1984). Adaptive control of mechanical impedance by coactivation of antagonist muscles. IEEE Trans Automatic Control.

[14] Lee A, Chadde M, Altenmüller E, Schoonderwaldt E (2014). Characteristics of task-specific tremor in string instrument players. Tremor Other Hyperkinet Mov (NY).

[15] Lee A, Tominaga K, Furuya S, Miyazaki F, Altenmüller E (2014). Coherence of coactivation and acceleration in task-specific primary bowing tremor. J Neural Transm.

